# Beta‐Thalassemia Major Complicated by Streptococcal Toxic Shock Syndrome: A Rare Case of Survival and Successful Management

**DOI:** 10.1155/crh/9991481

**Published:** 2026-02-08

**Authors:** Fajr Saeedi, Masaheer A. Aljehani

**Affiliations:** ^1^ Department of Pediatrics, Faculty of Medicine, King Abdulaziz University, Rabigh, Saudi Arabia, kau.edu.sa; ^2^ Department of Pediatrics, King Abdulaziz University Hospital, Jeddah, Saudi Arabia, kau.edu.sa

**Keywords:** beta-thalasemia, lymphadenopathy, septic shock, toxic shock syndrome

## Abstract

Beta‐thalassemia is an inherited blood disorder associated with defective hemoglobin production and impaired immunity, increasing susceptibility to severe infections. *Streptococcus pyogenes* poses a significant risk due to its potential to cause streptococcal toxic shock syndrome (STSS), a life‐threatening condition characterized by fever, shock, and multiorgan failure. We report a rare survival case of a nine‐year‐old child with beta‐thalassemia major who developed STSS secondary to *Streptococcus pharyngitis*. The patient presented with fever, neck swelling, hematemesis, and hypovolemic shock, requiring urgent resuscitation and intensive care unit admission. Laboratory tests revealed pancytopenia and renal dysfunction, and neck CT showed diffuse lymphadenopathy and fluid collection. A nasal discharge culture confirmed *Streptococcus pyogene* as the cause. The patient received empirical antibiotics, intravenous immunoglobulin (two doses), and supportive care, leading to clinical recovery. The neck swelling resolved, and ultrasound confirmed no residual fluid collection. This case highlights the critical need for early recognition and aggressive management in beta‐thalassemia patients with severe infections.

## 1. Introduction

Pediatric patients with thalassemia and hemoglobinopathies are at an increased risk of recurrent and potentially fatal infections [1]. Infections are the second leading cause of mortality in these patients, following heart failure [2, 3], and have been identified as the most common cause of death and morbidity in some studies, such as Wanachiwanawin et al. [4]. The heightened risk of infections is attributed to factors including repeated blood transfusions, which can lead to immune modulation and increased infection risk; invasive procedures such as central venous line insertion, splenectomy, or bone marrow transplantation; and nutritional deficiencies, particularly reduced zinc intake [1].

*Streptococcus*, a Gram‐positive coccus, is responsible for 1.8 million cases globally [5]. One of the most severe complications of streptococcal infections is streptococcal toxic shock syndrome (STSS), a life‐threatening condition characterized by hypotension, high‐grade fever, rash, desquamation, and multiorgan failure [6]. The mortality rate associated with streptococcal infections is considerably high, reaching up to 47% across all age groups [7] and approximately 5%–10% among pediatric patients [8]. Moreover, mortality in STSS is markedly elevated, with reported rates of 28% compared with 0% in non‐STSS cases [9]. Despite its clinical severity, data on the incidence of this condition in children remain limited [10–13], especially in Saudi Arabia, where the first pediatric case was reported in 1994 [14]. To date, no studies have specifically investigated in vulnerable populations such as patients with thalassemia.

We report a rare survival case of a nine‐year‐old female with beta‐thalassemia major who developed STSS, with pharyngitis identified as the primary site of infection caused by *Streptococcus* species. The patient initially presented to the emergency department with a sore throat, fever, and a large cervical lymph node resulting from streptococcal pharyngitis.

### 1.1. Case Description

A nine‐year‐old child, a known case of beta‐thalassemia major, presented to the emergency department with a three‐day history of progressively worsening fever and respiratory symptoms. She was previously diagnosed with the intron 1 position 110 guanine‐to‐adenine (intervening sequence *G* > *A*) homozygous mutation in the beta‐globin gene located on chromosome 11. This mutation followed an autosomal recessive inheritance pattern and was confirmed through hemoglobin electrophoresis and genetic testing. Initially, she was treated at a local clinic with azithromycin and paracetamol; however, her symptoms persisted over the next 3 days and were accompanied by snoring during sleep, though without signs of obstructive apnea. She subsequently developed multiple episodes of coffee‐ground vomiting and progressive drowsiness with altered consciousness, prompting urgent hospital admission. Her medical history included regular blood transfusions every 3 weeks for thalassemia, with no prior Pediatric Intensive Care Unit (PICU) admissions. She was on continuous treatment with Ferriprox, folic acid, and vitamin D, and her vaccinations were incomplete after the age of four. On arrival, the patient was in uncompensated septic shock. Her vital signs revealed hypotension (blood pressure 70/30 mmHg), tachycardia (140 bpm), and fever (39°C). She was drowsy with a Glasgow Coma Scale (GCS) score of 7/15, dusky lips, shallow breathing, and bilateral cervical lymphadenopathy. Both lymph nodes were tender and surrounded with erythema.

Laboratory tests indicated pancytopenia: white blood cells (WBCs) 0.27 × 10^9^/L, absolute neutrophil count 0.02 × 10^3^/μL, hemoglobin 6.3 g/dL, and platelets 91,000/μL. Coagulation studies showed prolonged prothrombin (PT) (18.5 s), aPTT (49.0 s), and INR 1.51. Renal function tests revealed mild azotemia, hyponatremia (Sdium levl = 126 mmol/L), normal potassium (4.1 mmol/L), and metabolic acidosis on venous blood gas analysis. Her C‐reactive protein (CRP) was markedly elevated at 233 mg/dL.

**Figure 1:**
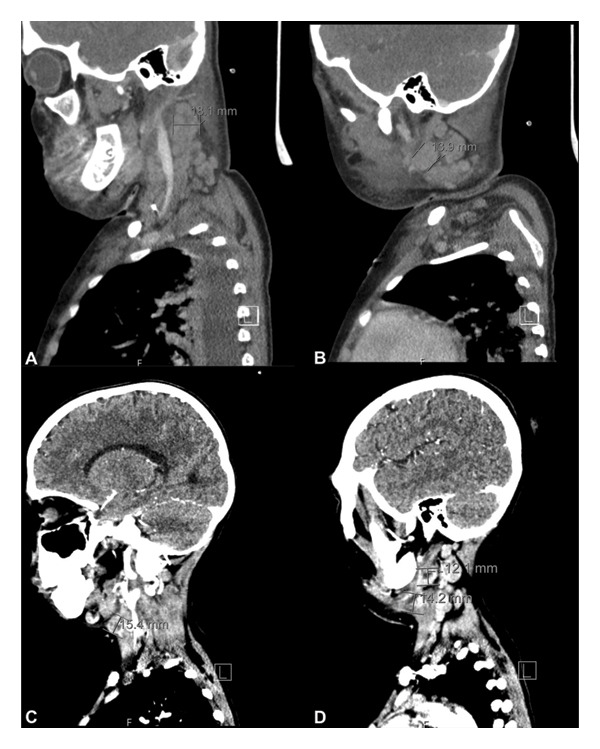
(A, B) Sagittal view CT scan with contrast showed scattered lymph node enlargement. (C, D) Follow‐up sagittal view CT scan with contrast showed scattered lymph node enlargement.

The patient’s clinical condition was consistent with uncompensated septic shock and multiorgan dysfunction involving the respiratory central nervous systems, along with pancytopenia. Immediate management has been initiated and included intravenous fluid resuscitation and mechanical ventilation. She was then transferred to the PICU and started empirically on vancomycin and meropenem. Initial imaging included neck and brain Computed topography (CT) scans showed unremarkable brain CT but extensive bilateral cervical lymphadenopathy affecting multiple neck spaces, with the largest node measuring 1.8 cm in short axis (Figures 1(A) and 1(B)). During her PICU stay, her condition remained critical, with worsening cervical lymphadenopathy. Additional laboratory evaluation including serological analysis showed pancytopenia without malignant cells on peripheral smear, positive Epstein–Barr virus (EBV), and parvovirus IgG antibodies. Hepatitis (A, B, and C) and cytomegalovirus were all negative. Nasopharyngeal culture yielded *Streptococcus pyogenes*, and respiratory serological analysis also detected human enteroviruses and rhinovirus.

**Figure 2:**
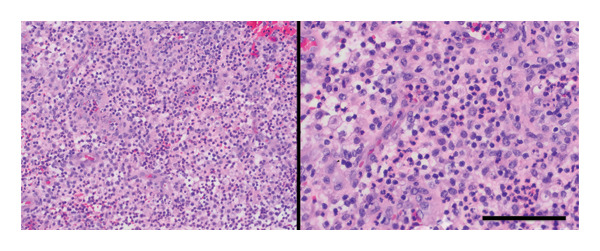
H&E sections in lower (left) and high (right) power field magnifications showed fibroadipose tissue with mixed inflammation and no evidence of malignancy, confirming the presence of a chronic abscess without morphologic or phenotypic features of lymphoma.

Despite broad‐spectrum antibiotics (vancomycin, meropenem, amikacin, and erythromycin) and antifungal therapy, she continued to have spiking fevers and severe neutropenia, necessitating intubation and inotropic support for 5 days. Follow‐up CT imaging showed persistent bilateral enlarged lymph nodes including retropharyngeal and upper jugular nodes (Figures 1(C) and 1(D)). After receiving two doses of intravenous immunoglobulin (IVIG), the patient showed remarkable clinical improvement within 2 weeks. Upon transfer to the ward, fever resolved and lymphadenopathy regressed. Excisional biopsy revealed necrotizing lymphadenitis with neutrophilic microabscesses, consistent with a chronic abscess; no lymphoma or fungal/acid‐fast organisms were identified (Figure 2). A final diagnosis of toxin‐mediated *Streptococcus pyogenes* lymphadenitis was made.

**Figure 3:**
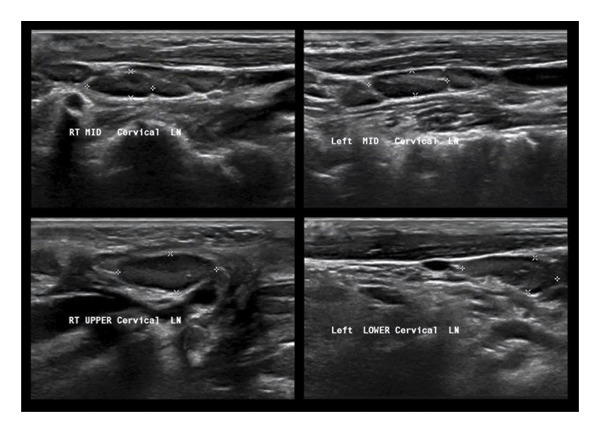
A follow‐up ultrasound imaging demonstrated normal findings with bilateral prominent cervical lymph nodes exhibiting preserved morphology, likely reactive to prior infection or inflammation, and no evidence of fluid collection.

After 30 days of hospitalization, the patient was discharged in stable condition. At follow‐up in 2 months, she remained well, with ultrasound confirming complete resolution of lymphadenopathy and no residual abscess formation (Figure 3).

## 2. Discussion

Although STSS has been reported in patients with alpha‐thalassemia, to our knowledge, this is the first reported case of such case in a child with beta‐thalassemia. The diagnosis of was based on clinical findings of shock, with blood pressure below the fifth percentile for age, multiorgan dysfunction including acute kidney injury, soft tissue necrosis, coagulopathy, and the identification of *Streptococcus pyogenes* in a nasal discharge culture. While the isolation of *Streptococcus pyogenes* from a nonsterile site raises some uncertainty about the diagnosis, as STSS typically requires isolation from sterile sites such as cerebrospinal fluid, blood, peritoneal fluid, or tissue biopsy [15], the diagnosis was further supported by excluding other causes through negative laboratory findings. Additionally, cervical lymphadenitis, fluid collections, and microabscesses observed on neck CT, confirmed through excisional biopsy, along with negative fungal and acid‐fast stains, provided strong evidence for the diagnosis.

STSS typically presents with fever, hypotension, and evidence of two or more organ systems’ dysfunction, such as hepatic, renal, coagulopathy, respiratory involvement, soft tissue necrosis, or erythematous macular rash. In most cases, the infection originates from the pharynx, as observed in our case, though it can also arise from the skin or an unknown source [13, 16]. TSS can be caused by *Streptococcus pyogenes* or *Staphylococcus aureus*. However, the predominant causative organism remains a point of debate in the literature. Some studies highlight *Streptococcus pyogenes* as the leading cause [13], while others suggest *Staphylococcus aureus* is more common [16, 17]. Despite this controversy, there is consensus that *Streptococcus pyogenes* leads to a more severe disease course, with higher risks of morbidity and mortality compared with *Staphylococcus aureus* [9, 16, 18]. Cook et al. (2020) further compared the two types of TSS, demonstrating that the streptococcal type is more common in younger patients and is associated with a more severe disease course. It results in longer hospital stays, increased capillary leakage, more pronounced respiratory involvement, and overall greater disease severity [19].

The severity of the disease is attributed to the secretion of exotoxins that enter the circulation and disrupt T‐cell activation, leading to an overproduction of cytokines and triggering an inflammatory cascade responsible for shock, fever, and multiorgan damage [20]. This process is particularly facilitated in immunocompromised patients, such as those with beta‐thalassemia, as seen in our case. Beta‐thalassemia is associated with impaired and diminished immunity, including a reduced CD4/CD8 ratio, impaired macrophage and neutrophil phagocytic activity, defective chemotaxis, decreased C3 and C4 levels, and reduced natural killer cell function [21, 22]. While immunoglobulin levels may be elevated in thalassemia, differentiation is impaired, contributing to immune dysfunction [21, 22]. These immune deficits arise from increased red blood cell hemolysis and the production of defective erythrocytes, which hyperactivate macrophages to phagocytose the defective cells, thereby reducing their capacity to combat pathogenic organisms [23, 24]. Additionally, severe anemia further increases the susceptibility to infections in thalassemic patients [1, 4]. Other factors contributing to the heightened infection risk in thalassemia include repeated blood transfusions, invasive procedures, splenectomy, bone marrow transplantation, and zinc deficiency, all of which are frequently observed in this patient population [1].

Early diagnosis and prompt management are critical to reducing mortality and morbidity. Supportive care remains the cornerstone of treatment and may include inotropic support, respiratory support, intubation, and kidney replacement therapies [13, 16, 25]. Antibiotic therapy is essential, with a recommended combination that provides coverage for both *Staphylococcus aureus* and *Streptococcus pyogenes*, as monotherapy is not advised [15]. First‐line options include first‐generation cephalosporins and beta‐lactamase‐resistant antistaphylococcal agents. Clindamycin has shown significant efficacy in many reports [13, 15, 19, 25]; however, it should not be used as monotherapy due to approximately 2% resistance in *Streptococcus pyogenes* [15]. Surgical debridement is another critical intervention in cases with localized infection, as it helps eliminate the infection source, reduces mortality, and can be lifesaving compared with medical therapy alone [26–28]. However, in our case, conservative treatment was chosen successfully because the patient only had microabscesses, and aggressive antistreptococcal antibiotics were initiated early due to a high suspicion of STSS. IVIG is also recommended in such cases, as it neutralizes superantigens, reduces cytokine secretion, and enhances bacterial clearance [15]. However, its effectiveness remains controversial. Cook et al. reported no significant differences in hospital stay or mechanical ventilation requirements between patients treated with IVIG and those who were not, a finding supported by several studies [19, 26, 29]. Conversely, Park et al. conducted a systematic review and meta‐analysis pooling data from five studies, showing that IVIG coadministered with clindamycin was associated with reduced mortality rates [30].

## 3. Conclusion

STSS is a severe, life‐threatening condition requiring early recognition, aggressive antibiotic therapy, and supportive management, including IVIG to neutralize bacterial toxins. Though IVIG’s benefit remains debated, early use may improve outcomes. In beta‐thalassemia, immune dysfunction and zinc deficiency heighten infection risk. Strengthening immune defense through nutritional optimization, particularly zinc supplementation, may reduce STSS susceptibility and enhance recovery, emphasizing the importance of comprehensive preventive and therapeutic strategies in vulnerable patients.

## Ethics Statement

Informed consent to publish has been obtained from the patient for this case report.

## Disclosure

FS and MA have critically reviewed and approved the final draft and are responsible for the content and similarity index of the manuscript.

## Conflicts of Interest

The authors declare no conflicts of interest.

## Funding

No funding was associated with this project.

## Data Availability

The data that support the findings of this study are available from the corresponding author upon reasonable request.
